# Fear of cancer recurrence in breast cancer survivors carrying a *BRCA1/2* mutation: A qualitative study

**DOI:** 10.1002/cam4.6889

**Published:** 2024-01-08

**Authors:** Josée Savard, Catherine Filion, Claudia Mc Brearty, Aude Caplette‐Gingras, Jocelyne Chiquette, Michel Dorval

**Affiliations:** ^1^ School of Psychology Université Laval Québec Québec Canada; ^2^ Centre de recherche du CHU de Québec‐Université Laval Québec Québec Canada; ^3^ Université Laval Cancer Research Center Québec Québec Canada; ^4^ Centre des maladies du sein, CHU de Québec‐Université Laval Québec Québec Canada; ^5^ Faculty of Medicine Université Laval Québec Québec Canada; ^6^ Faculty of Pharmacy Université Laval Québec Québec Canada; ^7^ CISSS de Chaudière‐Appalaches Research Center Lévis Québec Canada

**Keywords:** *BRCA1/2* genetic mutation, breast cancer, fear of cancer recurrence, psychological intervention, qualitative study

## Abstract

**Background:**

There is preliminary evidence suggesting that FCR is a major problem for breast cancer survivors carrying a *BRCA1/2* mutation. The goal of this qualitative study, conducted among women who were treated for breast cancer, was to provide a deeper understanding of how FCR is experienced in the context of a genetic predisposition to breast cancer.

**Method:**

Three focus groups (90–110 min) were conducted with 19 breast cancer survivors carrying a *BRCA1/2* mutation. The semistructured interview probed FCR level and impact, the role FCR played in the decision to have a prophylactic bilateral mastectomy and/or salpingo‐oophorectomy, the effect that surgery had on FCR, and the relevance of offering a psychological intervention targeting FCR to this population.

**Results:**

Findings indicated that FCR was a significant issue in these women, even though a majority had undergone a prophylactic surgery. Patients strongly affirmed the need to develop and provide access to FCR interventions that are specifically adapted to the needs of this group.

**Discussion:**

These results suggest that, although being the most effective medical option to reduce the actual risk of local recurrence (or second breast cancer), prophylactic surgery only partially reduces FCR. A psychological intervention targeting specifically FCR would be an appropriate complement to preventive surgery.

## 1 INTRODUCTION

The prevalence of *BRCA1/2* mutations varies from 2.7% to 7.8% in women with breast cancer and from 2.9% and 13.5% in those with ovarian cancer.^1^ Mutations in the *BRCA1/2* genes significantly increase the risks for breast and ovarian cancer. More specifically, the risk of developing breast cancer is greater than 60% and varies between 13% and 58% for ovarian cancer.^2^ In addition, breast and ovarian cancer survivors carrying a *BRCA1/2* genetic mutation are also at a greater risk of having a local recurrence or a second cancer. For instance, a study showed that the cumulative incidence of local breast cancer recurrence in *BRCA1/2* mutation carriers was 32% within 15 years after a breast‐conserving surgery (e.g., lumpectomy) and 9% after total mastectomy.^3^ Prophylactic interventions, such as unilateral or bilateral mastectomy and salpingo‐oophorectomy, can significantly reduce the risk of a first occurrence of breast cancer, as well as the risk of recurrence. Bilateral prophylactic mastectomy reduces breast cancer risk by 90%–95%.^4^ In addition, a meta‐analysis of women with a *BRCA1/2* genetic mutation found that a preventive salpingo‐oophorectomy leads to a significantly reduced ovarian cancer risk, ranging from 71% to 96%, and a 50% reduction in breast cancer risk.^5^

Although the risk of cancer recurrence following preventive surgery is significantly reduced, it is never null. Therefore, women will have to continue dealing with the uncertainty associated with a possible recurrence even after having undergone a prophylactic surgery. Fear of cancer recurrence (FCR) is defined as the fear, worry, or concern about the possibility of cancer returning or progressing.^6^ FCR affects almost all patients having been treated for cancer, to varying degrees. Indeed, FCR is one of the most reported concerns^7^ and was identified as one of the least met needs by patients^8^, ^9^ FCR can occur at any time during the care trajectory, at diagnosis, during and after treatment.^10^ Episodic FCR, which increases in the period surrounding medical tests and subsequently decreases when the results are negative, is considered normal. In other patients, FCR is clinically significant.^6^ Although other evolutions are possible, high/clinical FCR tends to remain stable over time in a large proportion of patients, even in those with a favorable cancer prognosis.^10^, ^11^

To the best of our knowledge, only one study has documented FCR in cancer survivors carrying a *BRCA1/2* mutation. This study was conducted by our research team among 89 breast cancer survivors.^12^ Results showed a mean score on the severity subscale of the *Fear of Cancer Recurrence Inventory* (FCRI‐S)^13^ of 16.8, which is above the cutoff score of 13 indicating a clinical level of FCR.^14^ In addition, 70.8% of the participants reported a clinical level of FCR, using the same criterion. This rate is higher than those obtained in previous studies of patients treated for cancer (e.g., breast, lung, pancreatic, and endometrial) also using the FCRI‐S, which ranged from 53.1% to 60.1%.^15^

The general aim of this qualitative study, conducted among women who were treated for breast cancer, was to provide a deeper understanding of how FCR is experienced in the context of a genetic predisposition to breast cancer. More specifically, the goals were to investigate: (1) how FCR manifests; (2) the role of FCR in the decision to undergo preventive surgery; (3) the effect that preventive surgery had on FCR; and (4) the relevance of offering a psychological intervention specifically targeting FCR for this population.

## 2 MATERIALS AND METHODS

### 2.1 Participants

The study included 19 women. To be eligible, participants had to meet the following criteria: (a) having been treated for a nonmetastatic breast cancer (stages I–III); (b) carrying a *BRCA1/2* mutation; (c) being between 18 and 80 years old; (d) being able to read and understand French; and (e) having Internet access. Women were excluded if they were on active cancer treatment other than adjuvant hormone therapy. Recruitment ceased when data saturation was reached. Participants were recruited among the 84 women who expressed a desire to receive a summary of the results of our previous study conducted in the same population.^12^ An invitation to participate in the current study was sent to them along with the summary of results. This recruitment  procedure was approved by the CHU de Québec‐Université Laval's ethics review board (#2022–5908). Twenty‐nine women initially answered positively to our invitation, of whom three were ineligible, five did not have common availabilities with other participants, and two did not provide their phone number for the eligibility interview.

### 2.2 Procedure

Women interested in the project were contacted by phone by a member of the research team to explain in more detail the goal and procedure of the study, the possible benefits and disadvantages, as well as to assess their eligibility. They were then asked to give their written consent via the REDCap interface. Participants were selected for focus groups using purposeful sampling, emphasizing variability on sociodemographic and medical characteristics (e.g., age, cancer stage, past curative and preventive treatments) to obtain a wide range of views and opinions.

Three focus groups (5–7 women per group) were conducted and recorded using the Zoom platform (duration from 90 to 110 min). They were led by two members of the research team previously trained for this purpose. A semistructured interview guide was developed beforehand to ensure that all themes were covered. First, participants were invited to summarize their trajectory of cancer diagnosis and treatments, genetic testing, and preventive surgery (when applicable). The following questions were about FCR (level, impact, and aggravating factors), the role it played in their decision to undergo a preventive surgery (when applicable), and the effect that genetic testing and preventive surgery had on FCR levels. Then, the relevance of offering a psychological intervention targeting FCR was discussed, as well as its possible content, the format in which it could be offered and when it should be offered in the cancer care trajectory (see Table 1 for questions). A payment of $75 was offered to compensate for the time spent on the study.

**TABLE 1:** Semistructured interview.

Topic	Questions
Genetic testing	When did you undergo your genetic test? Was it before or after your cancer diagnosis? What factors made you decide to undergo this test?
Preventive surgery	What decision did you make after receiving results of your genetic test with regard to the possibility of undergoing preventive (prophylactic) surgery? What type(s) of surgery did you undergo? Why this (these) one(s)? What factors made you decide whether or not you would undergo preventive surgery?
FCR	How do you cope with the risk of your cancer coming back? How often do you think about it? What is your current FCR level? To what extent is your FCR associated with distress and affects your daily functioning and quality of life? What factors increase or decrease your FCR level?
Genetic testing, preventive surgery, and FCR	To what extent was FCR a factor in your decision to undergo genetic testing and/or preventive surgery? What effect did the genetic test and/or preventive surgery have on your FCR?
FCR and interventions	What do you think of the idea of offering a psychological intervention to women who were treated for cancer and are carriers of a *BRCA1/2* mutation? What do you think of the idea of offering a psychological intervention that specifically targets FCR to these women? What do you think a psychological FCR intervention should address for this population? Is it important to offer an FCR intervention that is adapted to carriers of a *BRCA1/2* mutation (vs. an intervention that would include people with cancer in general)? Why so? What would be the specific intervention targets for this group? In what format should such a psychological FCR intervention be offered: group vs. individual vs. self‐administered (i.e., program completed by oneself at home; e.g., Internet‐based program)? What would be the benefits of offering an FCR group intervention? Individual? Self‐administered? What would be the disadvantages of each of these modalities? What would you think of a stepped care model that would start with a self‐administered intervention followed by an individual intervention offered to those still having high FCR after the first step? When should an intervention targeting FCR be offered?

### 2.3 Measures

#### 2.3.1 Sociodemographic and medical questionnaire

This questionnaire was developed to collect sociodemographic information including age, education level, marital status, employment, and income. It also gathered information on certain cancer characteristics (e.g., date of diagnosis and cancer stage), genetic testing (e.g., date and result), family history of cancer, and antecedents of preventive and curative cancer treatments.

### 2.4 Analysis

Descriptive statistics (frequencies and means) were obtained to describe the sample's demographic and medical characteristics. Focus groups were audio‐recorded and transcribed verbatim. Verbatim transcriptions were imported in NVivo 12.0,^16^ and a hybrid inductive‐deductive thematic analysis was performed.^17^ This method of analysis uses a thematic coding process that involves a balance between deductive coding (predetermined initial set of codes based on the interview guide) and inductive coding (themes that emerged from participants' discussions). Interviews were independently coded by two reviewers, that is the research coordinator (CF) and a doctoral student in psychology, and the analysis included five steps: (a) the development of the initial codebook; (b) synthesis of data and identification of initial themes; (c) application of the code template and insertion of additional codes; (d) grouping of codes and identification of themes; and (e) confirmation and substantiation of coded themes.^17^ Finally, the data from the thematic analysis was interpreted to give them meaning.

## 3 RESULTS

### 3.1 Participants' characteristics

Participants were on average 51.6 years old (see Table 2). The majority had a university degree (63.2%). Mainly, women were treated for a Stage I cancer (52.6%) and carried a *BRCA2* (57.9%) mutation. Most learned about their positive genetic mutation status after receiving their breast cancer diagnosis (78.9%). A large proportion of participants had undergone a preventive bilateral or unilateral mastectomy (73.7%) and a preventive salpingo‐oophorectomy (63.2%).

**TABLE 2:** Participants' demographic and clinical characteristics (*N* = 19).

Variable	Mean	Range
Age	51.6	34–76 (range)
	*n*	%
Education		
High school or junior college	7	36.8
University	12	63.2
Marital status		
Married/Common‐law relationship	15	78.9
Separated/Divorced	4	21.1
Occupation		
Full‐time job	11	57.9
Part‐time job	4	21.1
Retired	4	21.1
Income (CAD)		
0–60,000$	6	31.6
60,001–120,000$	8	42.1
120,001$ and more	4	21.1
Refused to answer	1	5.3
Stage of first breast cancer		
1	10	52.6
2	6	31.6
3	3	15.8
Treatments received (multiple responses for some participants)		
Intravenous chemotherapy	16	84.2
Radiation therapy	13	68.4
Hormone therapy (current)	3	15.8
Hormone therapy (past)	3	15.8
Oral chemotherapy	2	10.5
Brachytherapy	1	5.3
Second cancer/Recurrence (stage when applicable)		
No other cancer/recurrence	15	78.9
Second breast cancer	2 (1)	10.5
Recurrence in the same breast	1 (1)	5.3
Recurrence in contralateral breast	1 (2)	5.3
Cancer surgery (multiple responses for some participants)		
Total mastectomy	13	68.4
Partial mastectomy	9	47.4
Type of genetic mutation		
*BRCA1*	8	42.1
*BRCA2*	11	57.9
Prior knowledge of the genetic mutation status at the time of cancer diagnosis		
Yes	3	15.8
No	15	78.9
Reasons for oncogenetic consultation (several possible answers)		
To decide on upcoming treatments	7	36.8
To know their genetic status following the identification of a genetic mutation in the family	7	36.8
To know their genetic status due to family history of cancer	3	15.8
To find out their genetic status following a cancer diagnosis	10	52.6
Preventive mastectomy		
Bilateral	12	63.2
Unilateral	2	10.5
No mastectomy but considered in the future	2	10.5
No mastectomy and not considered in the future	3	15.8
Preventive bilateral salpingo‐oophorectomy		
Surgery undergone	12	63.2
Not undergone but considered in the future	6	31.6
Not undergone and not considered in the future	1	5.3

### 3.2 Summary of themes (see Table 3 for quotes)

**TABLE 3:** Quotes illustrating the main themes.

Themes	Patients' quotes
FCR as a decisive factor for undergoing prophylactic surgery	
Lower the risk of a cancer or cancer recurrence (main factor; *n* = 10)	“It was so that I could move on, to simply eliminate any risk of having cancer again.” “Given that I had an 85% chance it would come back if I didn't have my breasts removed, it was an obvious choice.”
Not to have to go through the experience (again; *n* = 5)	“I didn't want to live through this again, I didn't want to experience it all again, to go through the waiting, the stress, the constant tests.” “I wanted to be rid of my cancer and I never wanted to re‐experience this again if I survived my first cancer.”
Fear of recurrence (*n* = 5)	“I would say this was the number one reason [fear of recurrence].” “In my case it was really fear that made me agree to have my ovaries removed.”
Fear of dying (*n* = 3)	“I was totally terrified, I thought I was going to die at any moment.”
Frequency and severity of residual FCR after prophylactic surgery (*n* = 5)	“I think about it several times a week, but it isn't anything overwhelming.” “I'm still often afraid of a recurrence even if it's been 7 years now, there are a lot of triggers.” “I don't have this fear [cancer recurrence]. In fact, I only think about it when I have a pain somewhere or if I'm not well. Otherwise, it doesn't trouble me at all. On the contrary, I live in the here and now, intensely.”
FCR Consequences	
Negative scenarios (the most common effect; *n* = 4)	“I tell myself: Gosh, I'm really exhausted like this. What will it be like if I have to go through it all again? Then I imagine scenarios.” “Like it or not, you always have this fear, it'll come back as liver cancer, cancer here or cancer there, a brain tumor or bone cancer.”
Anxiety attack (*n* = 2)	“As soon as anything makes me think of it, that can trigger an anxiety attack.”
Sleep difficulties (*n* = 3)	“Every 6 months, I had the damned appointment and the week before I personally had trouble sleeping, you relive the whole process again.”
Avoid thinking in the long term (*n* = 1)	“I only think in the short‐term, sometimes medium term, but I never think about the long term, I don't allow myself that because of the fear of recurrence.”
Make the most out of life (*n* = 3)	“Honestly, for me the fear of recurrence has given a sense of urgency to my life.”
Factors increasing FCR	
Interpretation of physical symptoms (main factor; *n* = 12)	“I know that if I have a recurrence, it will be in some other part of my body, it won't be the breasts because I don't have them anymore. So it's clear that as soon as I have a slight symptom, whether in the head or somewhere else, one that lasts one or two days, or if it's unusual, I'll immediately start thinking about it, and that's when I can quickly descend into a state of panic.” “If I'm more tired or if I have a little ache, a pain, right away it's that thought that comes [recurrence].” “When I have a symptom I become a total hypochondriac, I wasn't like that before.”
Hearing about cancer (*n* = 5)	“If a friend of mine is diagnosed with cancer, one of my loved ones, or a friend of one of my friends, or one of my friends' friend's friends, it can be quite far removed, it doesn't have to be very close. If a celebrity dies of cancer […], I don't actually panic over it, but not far from that, I would say.” “When I see a TV program that talks about it [cancer], I think about it, but that's only a one‐off thing.”
Routine medical checkup/going to the hospital (*n* = 7)	“It's really hard for me, the MRI's every year, then the blood tests, the mammograms, then all sorts of things that at a certain point make going to the hospital stressful for me.” “When I go to the doctor […] Every six months I tell myself, “What will she tell me?” You could say, I don't know, that the hospital brings it all back again.”
Factors diminishing FCR	
Surgery (*n* = 9)	“It's been five years since my diagnosis, so soon five years of remission if you count from the end of the treatments. Right now I can say that my fear of a recurrence is mild, because I underwent all the preventive surgeries I could have at my age.” “After the genetic tests I took the decision to have preventive surgery for my ovaries and uterus. In fact, I told myself that I was stacking the odds in my favor and that really did a lot to reduce my worry about a recurrence.”
Medical tests (*n* = 3)	“I am being very closely monitored, so you could say that reassures us.” “I get an MRI every year for my breasts, I have a mammography every year, a checkup with my surgeon. You could say that in my head it's targeted, it's taken care of.”
Time elapsed since treatments (*n* = 6)	“I did a lot of research about my risk of recurrence, there are several types of breast cancer and mine is a triple negative, which means that it usually recurs very quickly. So, I know that after five years the risk is really lower. All that somehow reassures me.”
Busy life (activities and young children) (*n* = 4)	“It's really my kids, life goes superfast, it's back to nearly normal even in these times, I really have a lot of people around me and I'm lucky to have friends, a job I love, three kids with whom everything is great. Sure, when everything is going well in every part of life, then, of course, I don't think about it.” “I try to make the most of everyday life and that's what helps me not think about the fear. I'm always very busy, I always do a lot of activities.”
Short‐term effect of prophylactic surgery on reducing FCR	
Only reduces FCR over the short‐term (*n* = 9)	“It reduced my anxiety to have my cancer removed, then to remove something that might make me have to experience the same thing again, but the fear of recurrence didn't go away. Just the opposite, that's where it began, because when the treatments were over I told myself that it could come back somewhere else, so for me it didn't really have an impact on the fear of recurrence.” “It [surgery] really did a lot to reduce my worrying about a recurrence because I told myself “that's the game plan that seems the most appropriate to me”, so that was really good, but like I said, overwhelming fear came two years later when I got a message that they wanted to see me again, at the same time when my sister had her third cancer recurrence, that's what completely overwhelmed me.”
Relevance of offering an FCR intervention in breast cancer survivors carrying a *BRCA1/2* mutation	
Relevance (*n* = 16)	“I would say yes, based on the reaction I had when I got this offer to participate in today's Zoom meeting. It appealed to me and I'm happy that I did it, so that's why I say yes, I would be happy that something like this exists.” “Counselling, one does a course Genetics 101, something short in order to understand all this. You do the test, then you get the result and nobody really supports you when you're in this.” “I would like that, to have some support in our neck of the woods. A little bit everywhere in Quebec, not just in the big cities. That's essential.”
Talking with people who are going through the same thing (*n* = 7)	“I want to talk to people who experienced the same thing as me and ask them how they felt, to share with them and look for tools that help.” “It's good to talk with people who are going through the same thing, who are sort of in my age group, I find that also helps because it isn't the same situation as for my aunts for example.” “I think it would be important, not just people who've experienced cancer, but who have gone through the same testing process, with their family involved in this too, because most people haven't had this. So there still is a difference, there are specific things about this mutation that aren't in other cancers.”
Themes that should be included in psychological interventions offered to *BRCA1/2* mutation carriers	
Support in returning to work (*n* = 3)	“I had to quit my job, I made a career change, I simply resigned because I was no longer able to produce what they were asking of me at work, I wasn't at my full capacity. It brings a lot of impacts, so it's vital to have some support with psychologists or with a team and to help us get through this fear of recurrence.”
Body image and feelings of femininity (*n* = 3)	“I have my breast removed, that will transform me, maybe I'll no longer be the woman I once was, it's how others perceive you that changes.” “When you have a double mastectomy, how do you dress afterwards, swimsuits, etc. […]. At the Christmas party your colleagues, your girlfriends, your sisters‐in‐law all come wearing beautiful dresses with nice cleavage. I didn't do a reconstruction, so there you are in a high neckline and you try to hide it. All that is impacted, your life is impacted.”
Fear of recurrence in relation with genetic predisposition (*n* = 5)	“The fear of recurrence is very much connected to our genetic predisposition, so I think that it's after the announcement that you have the genetic mutation. For me it would make sense to get that support in connection with the fear of recurrence and our genetic predisposition.”
Guilt (*n* = 5)	“My feeling about it is that I felt guilty for having passed on that gene. That was a real worry for me to see how much it also worried them [family members].” “I had an aunt who had it, but she died because she found out about it too late. That's guilt, there are things that aren't my fault, but I feel guilty that I didn't speak about it soon enough, there needs to be support also in case other people [in the family] have it.”
Burden of being the message bearer to the family (*n* = 6)	“I was the one who brought that gift because the geneticist told me “that's a gift you're bringing your family”. I didn't see it like that, then afterwards I told myself, actually, yes it is […]. It was on my father's side, then it had a devastating effect in the family, it wasn't any fun. I had all that burden to tell them that it was because of me, then my son who will also have to do the test because I know he will want to do it because it's important, so he went through all that. It's me who is the bearer of news, of bad news, so it's a heavy burden to bear.”
Decision to have children (*n* = 2)	“A lot of things happening because I just had the treatments, I was leading an active life, making family plans and then there is the fear of a recurrence. I already had children. I was afraid to leave them, but I also wanted to have more so that means making a decision, then also the surgeries. […] I finally had another child, but that's it. Help in making a decision, but also help in managing all this at the same time.” “According to my doctor, I would be risking my life if I had another child, so that was a very difficult decision to make, to continue my pregnancy while asking myself “will I see him grow up”? There is also all that fear that was instilled into me like, “you won't have a child, it's too risky, the explosion of hormones will feed your cancer”. I had another child and I hope to see him grow up.”
Early menopause due to preventive surgeries (*n* = 4)	“With the next operation for ovaries and tubes, I am afraid of hormonal changes, mood changes, life changes, menopause.” “I didn't recognize myself anymore, because it actually triggered my menopause and I was still rather young and I didn't understand what was happening to me.”
Timing of psychological intervention targeting FCR	
Throughout the whole care trajectory and for a few years after the treatments (*n* = 16)	“I think it depends on the person. In my case it was when I went to take my test, I felt that it [the result] was going to be positive and that was difficult. For me that's the moment. For others it's when the results are announced. I found hearing the test results harder than I think the diagnosis of the cancer itself.” “I remember when my treatments were over I was leaving the hospital and then I saw there was this psychotherapy group on the fear of recurrence that was beginning in a few weeks at the same time I was going back to work, but I was feeling a little euphoric like “I've finished my treatments, I'm leaving the hospital, I never want to set foot here again, I don't need to, it's fixed, it's over, it's behind me”. But it's afterwards, when you're back to your normal life, when you have symptoms, when there are things that make you think about it and then you don't have any resources, you don't know who to turn to, or anything at all.” “I think what's important is that there should be some support and that maybe not everybody needs it at the same time. There are people who don't want to hear about it anymore, sometimes you avoid it, but every person has particular needs and at moments that might be very different. So, I think that the fact that there's help, that you know it's there, and that you can use it when you feel you need it, I think that would be important.” “Psychological support should be there from the beginning right to the end.”
Intervention format	
Self‐treatment over the Internet (*n* = 2)	“It's not always easy to find someone who is immediately available. I think it's good. In any case, I would appreciate it.” “I think it's really important that discussion groups exist, but I wouldn't go. That isn't for me, I love people, but I didn't want to hear about it anymore, […] I wanted to do that for myself and not in a group, I wanted to travel down that path alone.” “For me the stuff on the internet, the modules, that's really great. Also, I live in the country, we have discussion groups, but everybody knows everybody else because it's a small community. I went [to a group] and it's not for me. The information on a reliable website, yes, that's it.”
The importance of human contact (*n* = 6)	“I still think human contact is important. For me it's important to be with someone for that topic, the fear of recurrence, which affects you at the deepest part of yourself because ultimately it's about the fear of dying, so as far as I'm concerned, human contact is what's important.” “I think that before starting something on the internet, if there isn't any human contact, it's hard to trust. I mean, it could be anyone who is answering me on the other end of the email. I think if there were a meeting or some way you could know who you're dealing with, if there were some suggestions, strategies, if there were a little follow‐up, then that would be a lot easier. I'm not opposed to the internet, but I have a tough time starting something with a computerized system.”
Group (main intervention) (*n* = 10)	“I can tell you one thing that for me really helped the most, those were the meetings, it's not the one‐on‐one session with the psychologist because I did that too, but being surrounded by people who are experiencing the same thing, that's completely different.” “Everything is good, everything is important, but I think being in a group, brings a special dimension. When I had my first experiences, groups didn't work out for me, it wasn't offered with people my age going through the same thing, so I really had to go to a psychologist but later, that's when it began to be more enriching, to have things to share with people who were experiencing the same thing as me.”
Individual (*n* = 6)	“For me individual support, if that's possible. Not everybody is comfortable talking in front of others.” “Right now I would be able to do it [group intervention], but when I needed it, I wasn't able to speak because I was so emotionally caught up that it was hard for me to talk even to one person. So, I think individual sessions are also important. Once you've taken it all in, digested it and you've passed a stage, then going to a discussion group is possible. But there was a time when I would have needed individual sessions.”
By videoconference (*n* = 8)	“It's perfect on Zoom. That way you can access people who live elsewhere. It also increases the pool of women with different experiences.” “I find it really practical. I'm not sure I would have participated in this discussion group if it had been held in the hospital or some other place. […] I try to make the best use of time with three young children, I try to be productive. So, at home you're in a comfortable environment where you don't have to relive the traumas of the hospital […]. Every time I go back to the hospital, flashbacks of a lot of things, no fun, so I find home really perfect. More and more, everybody is able to use Teams or Zoom.”
Stepped care (*n* = 7)	“I would see it as a blend of the two. There are people whose approaches are much more self‐teaching, who will go and do the research by themselves. Then later, yes, you do the modules, the reading, I would see this like a pathway that would lead us to a guided discussion group or one with themes.” “Maybe begin with the modules, things you can do on the internet and after that move towards the group discussion with themes. It's exactly the same thing. I would have readily taken part if that had been available at the time, that's for sure.”

#### 3.2.1 FCR as a decisive factor for undergoing prophylactic surgery

Participants underwent preventive surgery(ies) mainly to reduce their risk of having cancer or having a cancer recurrence and to avoid (re‐)experiencing cancer. A few women also revealed that they had undergone prophylactic surgery mainly to reduce their FCR and their fear of dying.

#### 3.2.2 Frequency and severity of FCR

Only a few women mentioned having low FCR. The majority disclosed that they still experienced frequent (e.g., several times/week) and fairly high levels of FCR despite years that went by. However, most reported that it did not affect their daily functioning.

#### 3.2.3 FCR consequences

Several negative effects of FCR were identified including having negative scenarios about the future and, less frequently, panic attacks, sleep difficulties, and feeling they could no longer have long‐term goals. On a more positive side, some participants said that FCR led them to enjoy life more and gave them a certain urgency to live.

#### 3.2.4 Factors increasing FCR

The occurrence and persistence of somatic symptoms, such as pain and fatigue, were the main trigger factor identified. Medical and imagery tests, follow‐up, and hospital visits were also frequently mentioned. Hearing about people from their surroundings or public personalities who had received a cancer diagnosis or had died of cancer, as well as information in the media about cancer were other triggers mentioned.

#### 3.2.5 Factors diminishing FCR

Prophylactic surgery, especially when both bilateral mastectomy and salpingo‐oophorectomy were undertaken, importantly reduced FCR. While medical tests and visits were frequently mentioned as FCR triggers, a few participants reported that receiving negative test results had a reassuring effect. Other factors included a longer time since cancer treatment (e.g., 5‐year “remission”) and keeping oneself busy with childcare and activities.

#### 3.2.6 Short‐term/partial FCR‐reducing effect of prophylactic surgery

Participants were unanimous about the short‐term and partial effect of preventive surgery on reducing FCR.

#### 3.2.7 Relevance of offering an FCR intervention for breast cancer survivors carrying a *BRCA1/2* mutation

All participants mentioned that they would have liked to be better supported in the period surrounding their cancer diagnosis and positive genetic test. Although genetic counseling was considered helpful to understand their situation, they felt they were left alone to deal with it. They also said that an intervention that specifically targets FCR would be especially relevant and that it should be made widely available, not only for those living in large cities. Because participants spontaneously had group sessions in mind (see below), some considered it critical to gather people who share the same genetic status and age. A group intervention specifically developed and offered for this population was considered more appealing than an FCR intervention open to patients with all types of cancers and without a hereditary predisposition. Some said that they felt “weird” or that they did not belong when participating in group discussions or even being in hospital waiting rooms with other cancer patients. They further emphasized that the burden associated with their situation, the constant threat to their lives and the fact that many of them had young children made them a “special” group with specific needs that ought to be considered.

#### 3.2.8 Themes that should be included in psychological interventions offered to *BRCA1/2* mutation carriers

Several themes unrelated to FCR were mentioned including return to work, body image issues, surgery‐induced menopause, and guilt (to have transmitted their genetic mutation to their children, to have brought this “gift” into the family) and the burden to be the bearer of bad news. More specific to FCR, women expressed the view that their FCR was strongly related to their genetic predisposition and that any possible intervention should take that factor into account. Also, women of childbearing age at their cancer diagnosis deplored the lack of support they received to decide whether or not they should have (more) children. Specifically, they expressed that the fear of leaving their children motherless if cancer returned, as well as the possible impact of a pregnancy (hormonal changes) on their recurrence risk were themes they felt should be included in an FCR intervention.

#### 3.2.9 Timing of psychological intervention targeting FCR

Opinions about the ideal timing for offering an FCR intervention on this population were diverse. Critical time points included the period following the genetic testing/cancer diagnosis and after the termination of cancer treatment. However, participants concurred that it should be made available throughout the whole care trajectory and for years thereafter, given the long‐term psychological effects of being a cancer survivor with a genetic mutation.

#### 3.2.10 Intervention format

Most women expressed a personal preference for a group intervention. To be able to share their experience with other women who have similar characteristics (e.g., young age) and who are on the same journey, and to benefit from others' experiences were considered critical. Incidentally, for some women, having the opportunity to talk to other breast cancer survivors carrying a *BRCA1/2* genetic mutation was a strong incentive to participate in this study's focus groups. However, some shared the view that group interventions were not for them and stated a preference for an individual or a self‐administered intervention. Individual interventions were seen by some women as more appropriate at the beginning of the trajectory than support groups or group interventions in order to have the space to talk openly about their own experience. The greater availability of self‐administered interventions and videoconferencing was emphasized as an important advantage, especially for those living remotely. On the contrary, approximately one third of the participants highly valued human contact and said that they would have difficulties relying only on a web‐based intervention. Also, videoconferences were judged positively as way of making support more readily available. Finally, when asked about the relevance of a stepped care model to treat FCR in this population, beginning with a self‐administered intervention (e.g., web‐based) followed by an individual or group intervention, a few participants expressed a favorable opinion. Overall, participants highly valued the possibility of adjusting the level of intervention to patients' needs.

## 4 DISCUSSION

The goals of this qualitative study conducted among breast cancer survivors were to deepen our understanding of how FCR is experienced in the context of a *BRCA1/2* mutation and to ascertain these women's needs in terms of psychological support. Overall, FCR was found to be a significant issue in breast cancer survivors carrying a *BRCA1/2* mutation, even though most participants (84.2%) had undergone at least one type of prophylactic surgery. This is not surprising given that participants were a subsample of our larger quantitative study showing that 70.8% had a clinical level of FCR.^12^ While its possible anxiety‐reducing effect was among the top reasons for undergoing a preventive surgery, many participants revealed that the effect on FCR was real but short‐lived and partial. Indeed, worries about a potential cancer recurrence, although not incapacitating, were frequent (e.g., several times a week). This is in line with a prior prospective clinical trial that revealed persistent cancer worries following preventive salpingectomy with delayed oophorectomy or standard salpingo‐oophorectomy in *BRCA1/2* carriers.^18^

Triggers for FCR were numerous, consistent with existing FCR theoretical models^19^, ^20^, ^21^ and similar to what has been found in cancer patients without a hereditary predisposition.^22^ They included having new or persistent somatic symptoms, hearing about other people having received a cancer diagnosis or having died of cancer, and information transmitted in the media. The period prior to routine oncological exams and visits was identified as particularly dreadful (“They might find something wrong”). On the contrary, receiving negative results at tests and physical exams were indicated as particularly reassuring.

Study participants also identified negative consequences of FCR similar to their counterparts with no genetic mutation, including anxiety (e.g., negative scenarios). A few participants mentioned that FCR led to sleep difficulties. The relationship between FCR and sleep difficulties has not received much attention but negative scenarios and worries about a possible recurrence are quite likely to increase hyperarousal at bedtime, which is known to have a central role in the onset and maintenance of sleep impairments^23^, ^24^ On a more positive side, FCR was also seen as creating a certain urgency to live.

What is clear from our results is that prophylactic bilateral mastectomy and salpingo‐oophorectomy, although being the most effective medical options to reduce the actual risk of breast cancer recurrence, only partially reduce FCR. This suggests that a psychological intervention targeting specifically FCR would be an appropriate complement to preventive surgery. As pointed out by the study participants, such an intervention could be offered soon after the cancer diagnosis/genetic positive result but should be made available throughout the cancer care trajectory and even years after cancer treatment as FCR is likely to persist over time.

When asked about the relevance of offering an FCR intervention specifically to breast cancer survivors who are carriers of a *BRCA1/2* genetic mutation, women strongly affirmed their special needs particularly in relation to their younger age and greater risk of cancer recurrence, raised several unique issues (e.g., decisions about having children and possibility of leaving their children motherless). They also spontaneously talked about group sessions. This speaks to the importance they attributed to “human contact” and to being able to share with other people going through the same experience. It must be noted that they did not distinguish between support groups and group psychotherapy. Admittedly, the preference for group sessions may be due, at least in part, to a selection bias, since accepting to take part in a focus group probably indicates an inclination for group‐based approaches in general. A growing number of FCR interventions have been developed and tested over the past decade, especially cognitive‐behavioral therapy. A recent systematic review and meta‐analysis revealed that effects of these interventions, taken globally, were statistically significant but of a small magnitude.^25^ Interestingly, larger treatment effects were found for group‐ as compared to individually‐administered FCR interventions.

However, some women said they preferred other intervention formats including individual and self‐administered interventions, especially at first and for women from rural areas. Regarding self‐administered interventions, studies on FCR have shown mixed results. For instance, Akechi et al. tested a CBT‐based smartphone intervention which included problem‐solving therapy and behavioral activation in young breast cancer survivors.^26^ Results revealed a significant reduction in FCR levels. On the contrary, van Helmond et al. did not find any significant short‐ and long‐term effect of a self‐help training specifically targeting FCR.^27^, ^28^ However, positive results have been found when using a blended intervention, that is, a combination of online and face‐to‐face interventions. For instance, the Survivors' Worries of Recurrent Disease (SWORD) study, combining conventional face‐to‐face CBT with online sessions, found a statistically and clinically significant effect in reducing FCR levels at post‐treatment and at 3‐ and 15‐month follow‐ups, as compared to a standard treatment.^29^, ^30^ The addition of therapist time may help create a therapeutic alliance^31^ and reinforce motivation to initiate and maintain engagement in the process.^32^ This is in line with one participant's comment that she was not against receiving a web‐based program, but would find it hard to begin an intervention with a computerized system.

With regard to a stepped care approach to treat FCR, participants were not aware that this existed, but, after it was explained to them what it was, several expressed a positive opinion about the idea of combining a self‐administered intervention with a face‐to‐face intervention for those needing it. Overall, our findings suggest that there is no one‐size‐fits‐all format and emphasize the need to adapt the delivery of FCR interventions to each patient.

The study has some limitations. As already mentioned, a selection bias may have artificially inflated the interest in group interventions, but also the frequency and severity of FCR observed. Requiring internet access to participate in the focus group may also have introduced a selection bias. Further, the inclusion of highly educated females limits the generalization of findings.

This study suggests that it is critical to better screen FCR in patients carrying a genetic predisposition to cancer as they constitute a high‐risk group for experiencing frequent and persistent FCR. The FCRI is the most widely used self‐report scale in research^15^ and its 9‐item severity scale can be used to screen clinical levels of FCR. Shorter screening tools (e.g., one‐item questionnaire), that are easier to integrate in routine care, are also being developed.^14^ Clinical trials are also needed to assess whether existing FCR interventions are effective in patients with a genetic predisposition for cancer or if some adaptations are needed.

## AUTHOR CONTRIBUTIONS

**Josée Savard:** Conceptualization (lead); data curation (supporting); formal analysis (supporting); funding acquisition (lead); investigation (lead); methodology (lead); project administration (lead); resources (lead); supervision (lead); validation (lead); visualization (lead); writing – original draft (lead); writing – review and editing (lead). **Catherine Filion:** Conceptualization (supporting); data curation (lead); formal analysis (lead); investigation (supporting); methodology (supporting); project administration (supporting); supervision (supporting); validation (supporting); visualization (lead); writing – original draft (supporting); writing – review and editing (supporting). **Aude Caplette‐Gingras:** Conceptualization (supporting); funding acquisition (supporting); investigation (supporting); writing – original draft (supporting); writing – review and editing (supporting). **Jocelyne Chiquette:** Conceptualization (supporting); funding acquisition (supporting); investigation (supporting); resources (supporting); writing – original draft (supporting); writing – review and editing (supporting). **Michel Dorval:** Conceptualization (supporting); funding acquisition (supporting); investigation (supporting); methodology (supporting); resources (supporting); writing – original draft (supporting); writing – review and editing (supporting). **Claudia Mc Brearty:** Formal analysis (equal); writing – review and editing (equal).

## CONFLICT OF INTEREST STATEMENT

The authors report no conflict of interest.

## Data Availability

The dataset used and analyzed during the current study is available from the corresponding author on reasonable request.
